# Towards understanding vaccine hesitancy and vaccination refusal in Austria

**DOI:** 10.1007/s00508-020-01777-9

**Published:** 2020-12-11

**Authors:** Anja Bauer, Daniel Tiefengraber, Ursula Wiedermann

**Affiliations:** Institute of Specific Prophylaxis and Tropical Medicine, Medical University of Vienna, Vienna, Austria

**Keywords:** Health literacy, Children, Education, Rates, Attitude, Knowledge

## Abstract

In Austria, data on vaccine hesitancy is scarce. Available studies suggest around 1-11% of parents refuse vaccination, while many more are hesitant and consider refraining from some but not all of the recommended vaccinations. However, the key drivers for vaccine hesitancy in Austria are largely unknown. To learn more about vaccination coverage, attitude towards and knowledge around immunization as well as views on mandatory vaccination, we conducted a survey in a rural Austrian lay population including adults and children. Two paper-based questionnaires, one for adults 16 years or older and one for children aged 6-15 years, were developed, then sent to all houses of a rural community in Austria as well as handed out at the local primary and middle school, respectively. Self-reported coverage rates of children and adults were found to be low. Within the surveyed population 3% of children had never been or do not get vaccinated. More than half (57%) of the survey participants had a positive attitude towards vaccines, 21% were without reserves, 16% were found skeptical and 5% had a generally negative attitude. Knowledge about immunization in general was poor. Younger adults and people with secondary education appear to be most skeptical and negative towards vaccination. Children’s attitudes were closely linked to those of their parents. The major concern around vaccination in adults was fear of side effects. In adults, 54.2% support mandatory vaccination for Health Care Workers and 20.7% are against it. 39% of adults and 37% of children wanted more information on vaccination, preferably provided by physicians. Knowledge about disease prevention by vaccination should be improved and children could also benefit from an early age-appropriate vaccine education to strengthen health literacy. Physicians are the most trusted source of health information. Medical doctors should be aware of their very important role in transmitting trusted health information. This should include an up-to-date education in communicable disease prevention and immunization during their whole medical career. Furthermore, the curricula of health-care workers may need to be improved and harmonized concerning prevention and vaccination.

**Electronic supplementary material:**

The online version of this article (10.1007/s00508-020-01777-9) contains supplementary material, which is available to authorized users.

## Introduction

Vaccination is considered a safe, effective and cost-saving public health measure for disease prevention [1, 2]. Next to safe water, the impact of vaccines on mortality reduction and population growth is estimated to be larger than that of antibiotics and improvements in nutrition [3]. The success of global immunization programs has been impressively demonstrated by the dramatic decrease in morbidity and mortality of diseases, such as measles, polio, and tetanus [4].

Despite this success, today we face a global hesitancy and skepticism against vaccination, primarily in industrialized countries [5, 6], which correlates with the re-emergence of vaccine-preventable diseases, such as measles or pertussis. With the World Health Organization (WHO) goal of 95% measles vaccination coverage rate unmet, Europe faces a yearly increase in measles outbreaks. In 2019, 13,200 cases of measles were reported by 30 European Union (EU)/European Economic Area (EEA) member states with Lithuania (298.5/million), Bulgaria (176.4/million), and Romania (87.9/million) showing particularly high rates. The overall notification rate was 25.4 cases per million population, which was lower than in 2018 and 2017 (34.4 and 35.5 per million population, respectively), but much higher than the rates observed in 2015–2016 (7.8–9.0 per million population) in Europe. In Austria, 17.6 cases per million inhabitants (*n* = 151) were reported and 4 years earlier, in 2015, Austria had the second highest case-per million rate in all EU/EEA countries making up 36.0 cases/million with 309 notified cases of measles [7]. For pertussis, notified cases in Austria have risen steadily from 579 to 2231 between 2015–2019 [8]. Some European countries have recently introduced various forms of mandatory vaccination or extended their programs [9, 10]. Since then it has been a matter of debate whether such a strategy is applicable to all European countries, including Austria.

In 2014, the WHO Special Advisory Group of Experts (SAGE) on Vaccine Hesitancy defined vaccine hesitancy as “a delay in acceptance or refusal of vaccination despite availability of vaccination service. Vaccine hesitancy is complex and context specific, varying across time, place and vaccines. It is influenced by factors such as complacency, convenience and confidence.” Determinants include risk perception of vaccine-preventable diseases and necessity of vaccines, availability, affordability, willingness to pay and health literacy as well as trust in vaccine effectiveness, vaccine safety, health services, professionals and policy makers [11].

Another term, vaccine denier, refers to a member of a subgroup at the extreme end of the hesitancy continuum (between undoubtful acceptance and complete and undoubtful refusal); one who has a very negative attitude towards vaccination and is not open to a change of mind no matter what the scientific evidence says. A vaccine denier ignores any quantity of evidence provided and criticizes the scientific approach as a whole [12].

According to a survey performed in 2013, 4% of Austrian parents considered themselves vaccine deniers, and 57% said they were skeptical towards vaccination [13]. In another study conducted in an Austrian emergency department in 2012, 11.4% of people said they were vaccine deniers and 38.9% stated that they were skeptical [14]. In a representative sample of Viennese parents with children, 82.7% had a generally positive view about vaccination, but 25.1% refused at least 1 recommended vaccination for their child [15].

Recently, two EU-wide surveys on vaccine confidence and attitudes, one online and one with representative face-to-face interviews, were commissioned by the European Union. In the online survey for Austria, 70.5% of adult participants agreed that “vaccines are important for children to have” while 4.7% tended to disagree and 3.0% strongly disagreed with this statement [16]. In the face-to-face interviews, 71% of Austrians agreed that “it is important for everybody to have routine vaccinations” while 18% tended to disagree and 5% strongly disagreed with the aforementioned statement [17]. In a convenience sample of parents in 18 European countries, another study found a self-reported vaccine hesitancy in 33% of Austrian participants, undecided ones in 16%, and 51% not reporting to be vaccine hesitant [18].

In Austria, a surveillance system to monitor changes in vaccination coverage especially at a regional level is lacking. Since 2015, the official national vaccination coverage for measles and polio in Austrian children and young adults is estimated based on an agent-based computer-simulated model using documented administered vaccines and orders by pediatricians as well as sales numbers of vaccines by producers. Coverage for the recommended 2 doses with measles-mumps-rubella (MMR) vaccine is estimated at 82% for the 2‑5 year-old and 89% for the 6–9 year-old groups. The biggest deficit is estimated in the 19–30 years age group with a 2-dose coverage of just over 70% [19]. For polio immunization, this model suggests a significant delay for the third dose of the hexavalent vaccine in 30% of eligible children and 6.5% of completely unvaccinated individuals in the 5–9 year age group [20]. For adults older than 30 years mainly sales numbers of vaccines, which are not included in the state financed national vaccination program, are available. With this information, recently published estimates for the influenza vaccination rate of the Austrian population went down from 15.4% in the 2006/2007 season to 6.1% for the 2015/2016 season. In additional telephone surveys the influenza vaccination rate in people older than 60 years was determined at 14% [21]. As age distribution of vaccinated persons for other vaccinations is unknown, they provide no reliable estimate for vaccine coverage.

To increase vaccination coverage, it is important to understand the major drivers for a reduced vaccine uptake in general and vaccine hesitancy and vaccine refusal in particular, to be able to effectively counteract prejudices and fear by population-tailored information and improvement of the accessibility of vaccines.

### Aim

Addressing a rural Austrian population of adults and of children attending public schools, the aim of this study was to find out about:

1. self-reported vaccination rates
2. attitudes towards vaccination in general and mandatory vaccination
3. knowledge about vaccines and vaccine-preventable diseases
4. concerns about vaccines and vaccination and sources of information about these issues
5. preferred source and content of future information on vaccination.

### Ethics

The ethics committee of the Medical University of Vienna reviewed and approved the study with the vote number 1681/2015.

## Methods

### Study population

Within the framework of a larger healthy village initiative in Lower Austria (https://praevenire.at), one community (Pöggstall) was randomly selected for studying vaccination hesitancy as well as providing and testing concepts for a tailored information campaign. The community facilitated contact with the local schools to ensure high participation of children. The first step, the assessment of the present state of vaccination coverage, knowledge and attitudes, is reported here. The village, about 100 km from Vienna, had a population of 2416 in 2016. Between July and September of 2016, a cross-sectional survey was conducted. Two questionnaires were developed, one for adults from age 16 years (later referred to as the questionnaire for the adult population) and a separate questionnaire designed for children aged 6–15 years (later referred to as the children’s questionnaire), who attend the local primary or secondary/middle school. People aged 16–17 years were added to the adult population because the mandatory school age ends after nine school years (when most school children are 15 years old) and might thus not be reached in the study organized by the local schools.

### Questionnaires

An introductory text gave information about the study project, the aims of the questionnaire and the benefit of participation for the community. The anonymous questionnaire for the adult population included questions in the following sections:

- Demography (age, gender, education, occupation, nationality)
- Attitudes towards vaccination and mandatory vaccination, and whether they would recommend vaccination to their social environment
- Knowledge about vaccination in general, principal source of knowledge, and preferred source of information
- Concerns around vaccination
- Self-reported immunizations, knowledge of the Austrian National Vaccination Schedule (ANVS)
- Knowledge of specific disease prevention and vaccines, e.g. influenza and HPV.

The anonymous questionnaire for the children’s population included broadly similar sections but added a question on their parents’ attitude towards vaccination and did not ask about concerns around vaccinations.

The questionnaires were in German, the English versions can be found in the supplement S1 Appendix and S2 Appendix.

### Distribution of the questionnaires

A total of 1200 questionnaires for the adult population were sent out with the quarterly village newspaper to all households in Pöggstall (one each). The children’s questionnaire (*n* = 350) was handed out at the local primary and middle school by teachers to all children of all school years. Children were asked to voluntarily participate by filling it out at home and handing it in back at school. Both questionnaires were also put up at the local doctor’s office, the local pharmacy, and the community office, to which all the questionnaires, except for the ones collected by teachers, should be returned.

### Statistical analysis

Descriptive statistics were produced as numbers and percentages. Percentages not summing up to 100% for forced-choice questions are due to missing values. A knowledge score was calculated as the number of correct responses to the questions on vaccines and vaccination-preventable diseases, which included six possible correct answers in the questionnaire of the adult population (six single choice questions) and ten possible correct answers in the questionnaires for children (three single choice and two multiple choice questions). To compare responses between the various subgroups of age, gender, education, and knowledge (and parents’ attitude towards vaccination in children) a generalized linear model was applied with binomial counts and logit link. Variables were chosen based on previous studies. Open-ended questions were noted separately, and a list of the answers was compiled. Paraphrases were combined to obtain meaningful categories. Exponentials of parameter estimates and 95% confidence intervals were obtained that reflect odds ratios relative to the reference category. All calculations were done using IBM SPSS Statistics for Windows, Version 25.0 (IBM Corp., Armonk, NY, USA). *P*-values below 0.05 were considered significant.

## Results

After 3 months of collection, we received a total of 306 completed questionnaires from the adult population (response rate 26%) and 320 from the children’s population (response rate 91%). Questionnaires completed less than 75% were excluded from analysis (*n* = 6 adults, *n* = 0 children). Out of 300 of the respondents 5 were removed from the survey as they stated they were <16 years, leaving 295 for further analysis among the adults. Four children were excluded due to being <6 years, leaving 316 children for further analysis. We included the few >15-year-olds into the group 10–15-year-olds and renamed the group children aged 10+ years.

Demographic data of the adult and children group of respondents can be found in Tables 1 and 2.

**Table 1:** Demographic data of the surveyed adults

		Total (%)	Female (%)	Male (%)
Gender	–	*n* = 295	65.4 (*n* = 193)	34.6 (*n* = 102)
Age (years)	16–24	10.5 (*n* = 31)	64.5 (*n* = 20)	35.5 (*n* = 11)
	25–39	25.4 (*n* = 75)	66.7 (*n* = 50)	33.3 (*n* = 25)
	40–60	42.4 (*n* = 125)	72.8 (*n* = 91)	27.2 (*n* = 34)
	60+	21.7 (*n* = 64)	50.0 (*n* = 32)	50.0 (*n* = 32)
Education	PE	19.7 (*n* = 58)	72.4 (*n* = 42)	27.6 (*n* = 16)
	SLE	41.7 (*n* = 123)	57.7 (*n* = 71)	42.3 (*n* = 52)
	SHE	18.6 (*n* = 55)	63.6 (*n* = 35)	36.4 (*n* = 20)
	TE	18.0 (*n* = 53)	75.5 (*n* = 40)	24.5 (*n* = 13)
	Non-response	2.0 (*n* = 6)	83.3 (*n* = 5)	16.7 (*n* = 1)
Occupation	Unemployed	3.4 (*n* = 10)	60.0 (*n* = 6)	40.0 (*n* = 4)
	Training	6.4 (*n* = 19)	73.7 (*n* = 14)	26.3 (*n* = 5)
	Employed	55.9 (*n* = 165)	68.5 (*n* = 113)	31.5 (*n* = 52)
	Self-employed	8.8 (*n* = 26)	76.9 (*n* = 20)	23.1 (*n* = 6)
	Retired	21.4 (*n* = 63)	54.0 (*n* = 34)	46.0 (*n* = 29)
	Non-response	4.1 (*n* = 12)	50.0 (*n* = 6)	50.0 (*n* = 6)
Nationality	Austrian	94.2 (*n* = 278)	64.7 (*n* = 180)	35.3 (*n* = 98)
	Other	1.4 (*n* = 4)	100 (*n* = 4)	0
	Non-response	4.4 (*n* = 13)	69.2 (*n* = 9)	30.8 (*n* = 4)

*PE* primary education, *SLE* secondary lower education, *SHE* secondary higher education, *TE* tertiary education

**Table 2:** Demographic data of the surveyed children

		Total (%)	Female (%)	Male (%)
Gender	–	99.4 (*n* = 314)	48.1 (*n* = 152)	51.3 (*n* = 162)
–	No response	0.6 (*n* = 2)^a^	–	–
Age (years)	6–9	21.0 (*n* = 67)^a^	48.5 (*n* = 32)	51.5 (*n* = 34)
	10–15	76.1 (*n* = 240)^a^	46.9 (*n* = 112)	53.1 (*n* = 127)
	15+ (= 15–18)	2.9 (*n* = 9)	88.9 (*n* = 8)	11.1 (*n* = 1)
Education	Primary school	24.8 (*n* = 78)	46.2 (*n* = 36)	53.8 (*n* = 42)
	Middle school	72.3 (*n* = 228)^a^	48.0 (*n* = 109)	52.0 (*n* = 118)
	Grammar school	2.9 (*n* = 9)	77.8 (*n* = 7)	22.2 (*n* = 2)
	No response	0.3 (*n* = 1)	–	–

^a^*n* = 2 children did not state their gender, one of them was 6–9 years old, the other indicated to attend the middle school and was 10–15 years old

### Vaccination rate for common vaccines in adults and children

**Fig. 1:**
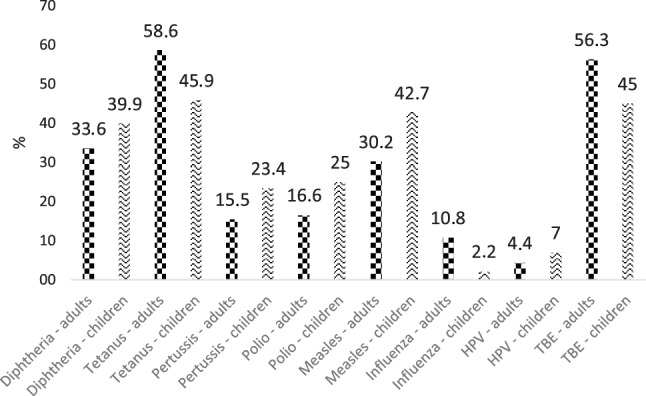
Self-reported vaccination rates in surveyed adults and children for selected vaccinations recommended in the Austrian National Vaccination Plan. *HPV* human papillomavirus, *TBE* tick borne encephalitis

As depicted in Fig. 1, a high percentage of adults and children reported a positive vaccination history towards tetanus, followed by TBE and diphtheria, whereas only few gave a positive feedback to pertussis vaccination. With respect to the desired 95% vaccination coverage rate against measles a concerning low percentage of adults and children reported a positive vaccination history.

Of the adults 24.7% reported being vaccinated against hepatitis B and 20.7% against hepatitis A. Only 5.1% of adults reported being vaccinated against pneumococci (PNC10/13, PPV23 not specified), and 1.0% against herpes zoster. Detailed results can be found in the supplementary Table S3.

Overall, 25.7% of children reported vaccination against hepatitis B, 7.6% against hepatitis A, and 11.1% against pneumococci. Of note, 3% of children explicitly and without having been asked remarked in an extra paragraph that they had never been vaccinated or do not get vaccinated. Detailed information can be found in the supplementary Table S4.

Regarding the HPV vaccination rate in adults, only 3.7% (*n* = 11, all female) reported a positive vaccination history. Of those women five said they had received all three vaccinations, one person said she had received one, and another five left that question unanswered. Most of the women vaccinated against HPV were between 25 and 60 years old. Female adults were also asked for their reasons for not having been vaccinated and offered multiple answers: 13% of them said the vaccination was not necessary, 9% said they were afraid of adverse reactions, 4% said because of costs, 3% said their doctor had advised against it, 2% said they were afraid of needles, another 2% said they missed the vaccination appointment, and 25% chose other reasons (5% nonresponse rate).

Concerning children, only 7.0% (*n* = 22) of 316 children said they were vaccinated against HPV, 17 female and 5 male, most of them aged 10 years or older (*n* = 21; *n* = 1 child 6–9 years old).

### Attitudes towards recommended and mandatory vaccination

#### Adults

##### Vaccinating according to national recommendations

When asked about their general attitude towards vaccination, 56.6% had a positive attitude, 21.0% claimed a neutral attitude, 15.6% were skeptical, and 5.4% had a negative attitude. Concerning age distribution, 67% aged 60+ years, 58% aged 40–60 years, 51% aged 25–39 years, and 45% aged 16–24 years viewed vaccination as positive. Concerning education, 73.6% with tertiary education, 52.8% with secondary higher education, 49.6% with secondary lower education, and 58.6% with primary education viewed vaccination as positive. Those with a skeptical or negative attitude towards vaccination were less likely to score higher on the knowledge score (odds ratio [OR] 0.63, 95% confidence interval [CI] 0.50–0.79), compared to people with a positive attitude, while no statistically significant differences concerning knowledge about vaccination were found for age, gender, and education.

In total, 55.6% of adults would recommend vaccination to their social environment, while 37.6% stated they would not. Those willing to recommend vaccination showed an OR of 1.66 (95% CI 1.39–2.00) for higher knowledge score. Those with secondary higher education were less likely to recommend vaccination (OR 0.34, 95% CI 0.14–0.82) compared to people with tertiary education (OR 1.0, reference category), while no difference between the latter and people of primary and secondary lower education was found. No statistically significant effects on the likelihood of recommending vaccines were found for age and gender.

Overall, 73.2% answered affirmatively when asked whether they would get their children vaccinated or whether they have had their children vaccinated according to the current ANVS “Impfplan Österreich 2016” [22], while 20.0% denied it. No statistically significant correlation was found for age, gender, education, and knowledge score.

##### Mandatory vaccination

Among adults, 39.3% agreed to a possible introduction of mandatory vaccination for attending state-operated facilities, such as schools, 34.2% did not agree, and 25.4% were undecided. With 60+ years old as reference category, people aged 40–60 years were less likely to agree to mandatory vaccination (OR 0.51, 95% CI 0.26–0.99), as were people aged 25–39 years (OR 0.46, 95% CI 0.22–0.97), while people aged 16–24 years were the least likely to agree to mandatory vaccination (OR 0.17, 95% CI 0.05–0.55). Those who agreed were more likely to score higher on the knowledge score (OR 1.46, 95% CI 1.24–1.73). No statistically significant differences concerning approval of mandatory vaccination were found for gender and education.

While 54.2% of adults were in favor of general mandatory vaccination for healthcare workers in hospitals and at doctor’s and midwifery practices, 20.7% were against, and 23.7% were undecided. Using tertiary education as the reference category, people with secondary higher education (OR 0.39, 95% CI 0.17–0.91) and people with secondary lower education (OR 0.41, 95% CI 0.19–0.85) were less likely to agree to mandatory vaccination for HCWs. Those who agreed were more likely to score higher on the knowledge score (OR 1.36, 95% CI 1.16–1.60). No statistically significant difference for people with primary education was found.

Detailed results can be found in the supplementary Table S5.

#### Children and their parents’ opinion

When asked about their general attitude towards vaccination, 47.4% of children answered having a positive attitude towards vaccination, 34.5% had a neutral opinion, 10.4% of children said they were rather skeptical and 7.0% were negative. Younger children aged 6–9 years were more likely to be of a skeptical or negative opinion (OR 2.51, 95% CI 1.04–6.05) compared to children aged 10+ years. Children with a skeptical or negative attitude were less likely to score higher on the knowledge score (OR 0.77, 95% CI 0.66–0.91). No statistical difference was found for children’s gender.

Regarding their parents’ opinion, 57.0% of children answered their parents had a positive opinion about vaccination, 23.4% claimed their parents had a neutral opinion, 10.8% said they were rather skeptical and 7.6% said their parents had a negative opinion concerning vaccination. Children who claimed their parents thought positively of vaccination were unlikely to have a skeptical or negative opinion themselves (OR 0.04, 95% CI 0.02–0.09), compared to children with parents with a skeptical or negative attitude. Female children were more likely to say their parents were of a skeptical or negative opinion (OR 2.09, 95% CI 1.16–3.78). No statistical difference was found for children’s age.

##### Vaccinating according to national recommendations

Overall, 63.0% of children thought they had received all the scheduled vaccinations recommended in the ANVP “Impfplan Österreich 2016” [22], while 33.5% of children answered they had not. Children claiming their parents had a positive opinion of vaccination were more likely to say they had received all scheduled recommended vaccinations (OR 3.86, 95% CI 2.02–7.37), compared to children who said their parents had a skeptical or negative attitude. Children who believe they had received all scheduled vaccinations were more likely to score higher on the knowledge score (OR 1.28, 95% CI 1.14–1.43). No statistically significant effects were found for children’s age or gender.

##### Mandatory vaccination

Of the children 30.7% agreed to vaccine mandates prior to attendance of kindergarten or school, 49.4% did not agree and 19.6% were undecided. Children reporting their parent’s opinion about vaccination being positive were much more likely to agree to mandatory vaccination for the attendance of kindergarten or schools (OR 13.33, 95% CI 3.15–56.42), compared to children who believe their parent’s opinion to be skeptical or negative. Children who agreed to the introduction of mandatory vaccinations were also more likely to score higher on the knowledge score (OR 1.16, 95% CI 1.03–1.30). No statistically significant difference in opinion about mandatory vaccination was found for children’s age or gender.

Among children, 40.2% approved mandatory vaccination for healthcare workers, 20.6% disapproved and 38.6% were undecided. Children who thought their parent’s opinion about vaccination to be positive were much more likely to agree to mandatory vaccination for HCWs (OR 6.39, 95% CI 2.58–15.84), compared to children who believe their parent’s opinion to be skeptical or negative. Children who agreed to the introduction of mandatory vaccination were also more likely to score higher on the knowledge score (OR 1.28, 95% CI 1.14–1.44).

Detailed results can be found in the supplementary Table S6.

### Subjective comprehension of vaccination and knowledge about vaccine-preventable diseases and vaccinations

Adults were asked four questions about people’s understanding of vaccination, followed by six questions on vaccine-related knowledge of measles, HPV and their respective vaccines (Table 3). A knowledge score was calculated as a number of correct answers for the six questions. Table 4 shows ORs and 95% CIs of scoring one point or more on the knowledge score by age gender and education. A higher age, female gender, and tertiary education were positively associated with points on the knowledge score.

**Table 3:** Responses of adults to questions about difficulties concerning decisions about vaccinations and knowledge about different vaccination issues

“How easy/hard is it …”	Very easy /easy (%)	Hard/very hard (%)	I don’t know (%)
“… to understand why you need vaccinations?”	62.0	24.5	8.1
“… to assess if information on health hazards in the media is reliable?”	23.3	60.4	10.2
“… to assess which vaccinations you might need?”	44.4	40.4	9.5
“… to decide if you should get vaccinated against influenza?”	55.3	30.5	8.1
*Knowledge items (correct answers in bold)*	*Yes*	*No*	*I don’t know*
“It is mostly children who fall ill during current measles outbreaks”	18.0	**31.2**	48.8
“The elimination goal for measles by the WHO was 2015”	**13.2**	4.4	78.6
“Measles could be eliminated via two doses of the MMR-vaccine and vaccination coverage of 95%”	**37.6**	6.4	52.9
“The risk of encephalitis through measles is about 1 in 1000 diseased”	**17.3**	7.1	73.2
“Two doses of the measles vaccine lead to lifelong protection”	**37.3**	15.3	44.1
“Human papillomavirus (HPV): the vaccine protects from the common HPV types, which may lead to cervical cancer or genital warts.”	**42.0**	5.8	45.8

Percentages of correct answers in *bold*

**Table 4:** Odds ratios (OR) and 95% confidence intervals (CI) for higher knowledge score (>1 correct answer) by sociodemographic attributes of adults

Variable	Category	OR	95% CI
Age (years)	16–24	0.39	0.19–0.77
	25–39	0.52	0.30–0.88
	40–59	0.98	0.60–1.60
	60+	1.0	–
Gender	Female	1.53	1.03–2.25
	Male	1.0	–
Education	PE	0.29	0.16–0.51
	SLE	0.43	0.26–0.72
	SHE	0.50	0.28–0.92
	TE	1.0	–

*PE* primary education, *SLE* secondary lower education, *SHE* secondary higher education, *TE* tertiary education

Children were asked the same four questions as the adults to learn about their subjective understanding of vaccination (Table 5), followed by five questions (two out of five multiple choice questions) with a total of ten correct answers on their vaccine-related knowledge. Again, a knowledge score was calculated as correct answers out of ten. Table 6 shows ORs and 95% CIs for a higher knowledge score, which were positively associated with a positive parents’ opinion on vaccination.

**Table 5:** Responses of children to questions about difficulties concerning decisions about vaccination

“How easy/hard is it …”	Very easy /easy (in %)	Hard/very hard (in %)	I don’t know (in %)
“… to understand why you need vaccinations?”	72.1	14.2	12.7
“… to assess if information on health hazards in the media is reliable?”	21.8	41.7	33.2
“… to assess which vaccinations you might need?”	37.4	40.9	20.9
“… to decide if you should get vaccinated against Influenza?”	45.8	29.1	23.4

**Table 6:** Children’s odds ratios (OR) and 95% confidence intervals (CI) for higher knowledge score (>1 correct answer) by sociodemographic attributes and parents’ opinion

Variable	Category	OR	95% CI
Age (years)	6–9	0.91	0.49–1.72
	10–15+	1.0	–
Gender	Female	0.81	0.48–1.36
	Male	1.0	–
Parents’ opinion	Positive	3.85	1.97–7.55
	Skeptical/negative	1.0	–

### Concerns around vaccination

Participants in the adult group were asked to describe the concerns they had towards vaccination as an open question. Overall, 59.7% answered this question. Answers were categorized into concerns about side effects (*n* = 114; 38.6%), 12.5% (*n* = 37) said vaccinations were not important or unnatural, 5.4% (*n* = 16) said they were potentially harmful to the immune system, 4.4% (*n* = 13) said they objected to the money-driven pharmaceutical industry, 4.1% (*n* = 12) were concerned about vaccine ingredients and 1.4% (*n* = 4) objected to the practice of multiple vaccinations. Further 12.5% (*n* = 37) named concerns or made statements that could not be as easily categorized, such as some were concerned that panic is spread (e.g. avian influenza in 2009/2010) to sell medication or vaccinations, some believe that the number of vaccines in the vaccination schedule cannot be good for their children, that the costs were too high to afford all the recommended vaccines, and that potentially massive damage could be done to the human body through vaccination.

As a specific example, adults were asked what they regarded as the primary reason for refusal of the influenza vaccination in Austria. Among the multiple answers given, 37.6% chose afraid of side effects, 20.7% said the vaccination makes me ill, another 18.0% said it was the ineffective protection, 9.2% said I am not at risk, 11.9% chose other reasons.

Children were not asked for concerns regarding vaccination.

### Source of information and content

**Fig. 2:**
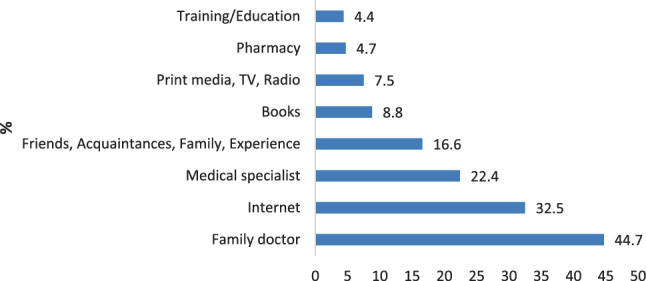
Sources of information on vaccination reported by adults (multiple answers possible, 19.3% non-response rate)

**Fig. 3:**
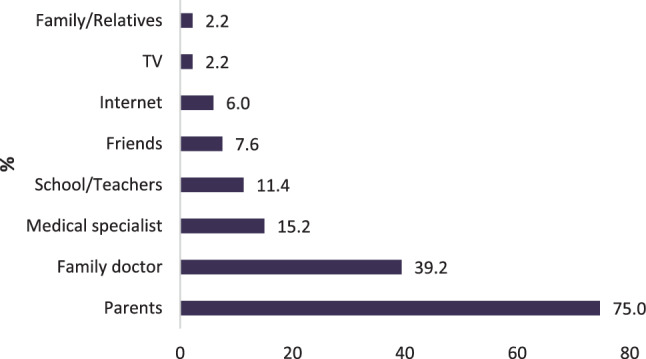
Sources of information on vaccination reported by children (multiple answers possible, 8.2% non-response rate)

Most adults named the family doctor (44.7%) as their source of information on vaccination. Among children, 75.0% named their parents as their source of information about vaccination. Figs. 2 and 3 show detailed results.

**Fig. 4:**
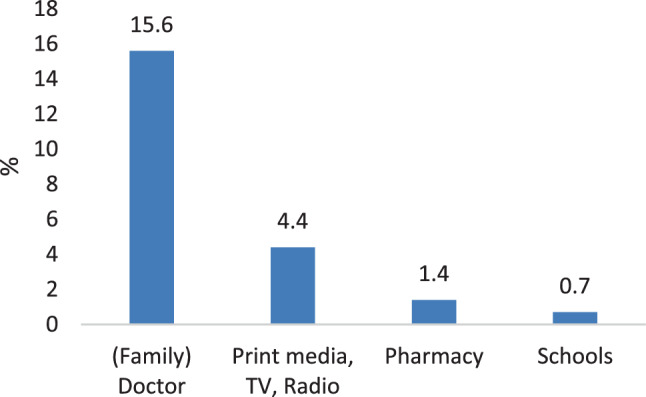
Preferred future sources of information by adults

**Fig. 5:**
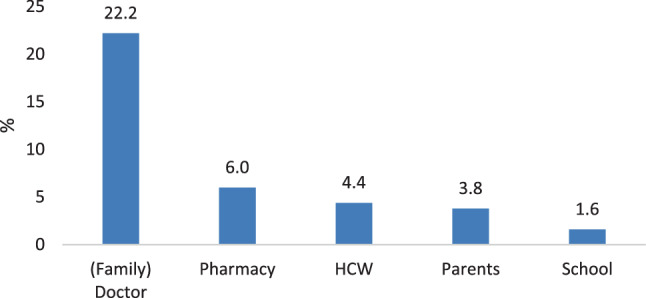
Preferred future sources of information by children. *HCW* health care workers

Further information about vaccination was preferred in 38.6% of adults and 37.3% of children. Both groups specified the family or specialist doctor as their preferred future source of information. See Figs. 4 and 5 for detailed results.

**Fig. 6:**
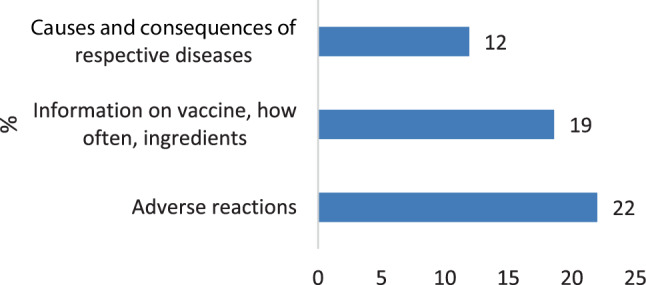
Future information desired by adults

**Fig. 7:**
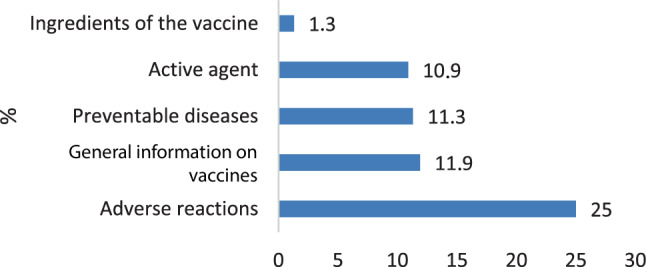
Future information desired by children

The majority in both groups (22% of adults and 25% of children) wanted to receive more information about adverse reactions in the future (*see* Figs. 6 and 7).

## Discussion

This survey provides information on attitudes and knowledge about vaccination along with self-reported vaccination rates in children and adults of a small Austrian village. We could identify the most trusted sources of information and important reasons for concerns towards vaccination.

The high response rate in the group of children up to 16 years (320 out of 350 questionnaires or 91%) offers valuable information on the attitudes and knowledge about vaccination to tailor educational programs to the needs of this generation.

Our results showed a moderate self-reported vaccination coverage for TBE and tetanus, low coverage of measles, mumps, rubella, diphtheria, pertussis and polio and very low vaccination coverage for influenza and HPV in adults and children. Of the children 3% reported they had never been vaccinated or do not get vaccinated, which seems to be in line with studies of vaccine refusal in western Europe [17, 23]. The generally low self-reported coverage rates could either suggest a substantial lack in many essential vaccinations and/or a poor knowledge of their own vaccination status. Some authors have attributed this lack of awareness in the general population to a lack of social marketing: preventive measures cannot be successful unless the tools of modern communication sciences are put to full use. In Austria, there has been insufficient vaccination promotion activity in the past, and the stakeholders have not been able to agree on a common approach [24]. Furthermore, while it is well known that financial reimbursement and the free supply of vaccines are important factors for increasing vaccination rates [25], self-funding is still the norm for adults in Austria, and with the exception of the MMR vaccine no general financial reimbursement has been implemented for immunizations.

While the general attitude towards vaccination was positive in two thirds of people aged 60+ years, this dropped to less than half in people aged 16–24 years. Older people may have personal experience with certain vaccine-preventable diseases and therefore value disease prevention higher than younger people [26]. A large survey of Italian pediatricians found an advantage in vaccine knowledge and confidence in older professionals [27]. Furthermore, older people might have higher trust in their physician due to more frequent consultation for other health problems.

In adults, tertiary education appeared to be correlated with a positive attitude towards vaccination, while people of secondary higher education showed a trend to have the most skeptical views, although the differences were not statistically significant. We found people of secondary higher education to be least likely to recommend vaccination to their social surroundings. These findings are not completely in line with other research, which found a high educational and socioeconomic level as a marker of vaccine acceptance for themselves and their children [16, 17, 28] or no effect of these variables [29].

In recent years, mandatory vaccination was introduced or expanded in several European countries and came with some protests of the respective public [30]. While compulsory vaccination is not envisaged by the government for the general population in Austria, but a matter of consideration for healthcare professionals, it is of value for public health policy makers to learn that more than half of the adults (54.2%) in our survey support mandatory vaccination for HCWs and only one in five (20.7%) are against it.

Regarding adult’s and children’s subjective comprehension of immunization, many children and even more adults in our study had trouble understanding why they needed vaccinations. Most of them found it especially hard to consider the quality of information concerning health hazards in the media and found it hard to understand which vaccinations they personally needed. Our adult population showed only limited knowledge when it came to measles and HPV, and many children stated they did not know how vaccines worked. A recent EU-wide survey found a high variability in vaccine knowledge with Austrians ranked around the EU-average. A considerable difference in knowledge between subjective social classes (self-defined upper class vs. working class) has been observed at the EU-wide level [17].

Regarding the major source of information, we confirm the physician as the most important contact person for adults to deliver information about and build trust in vaccinations, as has been shown extensively in other research [15, 17, 28, 29, 31]. The majority of children (75.0%) named their parents as influential source of information about vaccination, but a significant percentage (39.2%) also valued their family doctor.

Our study offers further insights about what kind of information people want from their physician and shows that doctors have a chance in delivering important messages on vaccination before people seek information from other sources, especially online and print media.

It appears that healthcare professionals need to become more aware about their significance as role models and source of trusted and valued information. Greater efforts to support health education and physician training are needed to give tailored vaccine information allowing a sound and well-informed decision by their clients and patients. Our study shows that also children could benefit from an early age-appropriate vaccine education to strengthen their health literacy.

### Limitations

Paper-based surveys or telephone-based surveys are valuable measures in public health epidemiology; however, our questionnaires were not validated and therefore we cannot quantify how accurately they measure the endpoints. The adult questionnaire showed only a limited response rate and two thirds of the responders were women. It is also unknown how many of the surveys were completed at the doctor’s office, the pharmacy, or the community center, where they were also available (in terms of response rate). Concerning the lay population, we did not ask specifically whether people were employed in the healthcare sector. Regarding vaccination coverage, self-reported numbers of past vaccinations do not necessarily mirror the actual vaccination coverage.

As we correlated knowledge with opinion, it needs to be noted that some children with a skeptical or negative attitude towards vaccination might have purposely answered in the negative when being asked whether vaccination is important to be protected from possibly severe diseases or if vaccination helped their own body’s defenses to be protected later on by learning about sickness-causing triggers, as they or their parents might not trust the scientific basis of these established facts, despite abstract knowledge of them. The same could be true for adults being asked about the measles and HPV vaccine.

## Conclusion

In Austria, studies on determinants of vaccine hesitancy are scarce. In our survey, self-reported coverage rates children and adults were found to be low and could either suggest problems with vaccine uptake and/or a poor knowledge of vaccination status. Of the children 3% reported they had never been vaccinated or do not get vaccinated.

The general attitude towards vaccination was positive in two thirds of adults aged 60+ years, but this dropped to less than half in people aged 16–24 years. Adults with a secondary higher education were least likely to recommend vaccination to their social surroundings. More than half of the adults (54.2%) supported mandatory vaccination for HCWs and one out of five (20.7%) were against it.

We could confirm the physician as the most trusted source of information around vaccination in adults. Greater efforts by healthcare professionals are needed to give tailored vaccine information, allowing a sound and well-informed decision. Doctors should be aware of their very important role in transmitting trusted healthcare information. This should include an up to date education in communicable disease prevention and immunization during their whole medical career.

In Austria, more research regarding determinants and state of vaccine hesitancy is needed to be able to implement evidence-based strategies for improvement of vaccination coverage and disease prevention by vaccination.

## Caption Electronic Supplementary Material

S1 Text. Towards understanding vaccine hesitancy. Questionnaire for the adult population

S2 Text. Towards understanding vaccine hesitancy. Questionnaire for the child population

S3 Table vaccination rate of individual vaccines in surveyed adults, as well as OR and 95% confidence interval of having been vaccinated by age, sex, education, and knowledge score

S4 Table vaccination rate of individual vaccines in surveyed children, as well as OR and 95% confidence interval of having been vaccinated by age, sex, parental skepticism, and knowledge score

S5 Table OR and 95% confidence interval of skeptical and negative attitude/vaccine recommendation/getting one’s children vaccinated/agreement to mandatory vaccination for state-operated institutions/agreement to mandatory vaccination for HCW by age, sex, education, and knowledge score

S6 Table OR and 95% confidence interval of a general skeptical or negative attitude/all vaccinations received/agreement on mandatory vaccination school attendance/agreement to mandatory vaccinations for HCW in children by age, sex, parents’ opinion, and knowledge score
