# Supplementary material for: Towards understanding vaccine hesitancy and vaccination refusal in Austria
Source: Wien Klin Wochenschr. 2020 Dec 11;133(13-14):703–13. doi: 10.1007/s00508-020-01777-9 (PMC8292253; doi:10.1007/s00508-020-01777-9)
Supplement: Supplementary file 1 — S1 Text. Towards understanding vaccine hesitancy. Questionnaire for the adult population [file 508_2020_1777_MOESM1_ESM.docx]

Questionnaire for the adult population

Introduction: Information on the project in Pöggstall and the survey

**"**This questionnaire is designed to gather vaccination status, knowledge, and opinions regarding vaccination from citizens in Pöggstall. It is part of the project „PREVENT TOGETHER IN PÖGGSTALL“. Please answer as many of the questions as possible. Thank you very much!“

1. Questions about yourself

1.1 Age

- 6-15 y.
- 16-24 y.
- 25-39 y.
- 40-60 y.
- > 60 y.

1.2 Sex

- female
- male

1.3 Education

- primary education
- apprenticeship/vocational school
- Matura (Higher School Certificate)
- tertiary education

1.4 Employment status

- unemployed
- in training
- a company employee
- self-employed
- retired

1.5 Nationality

- Austrian
- Other: _______________

2. General questions on vaccinations

2.1 How would you describe your general attitude towards vaccination?

- positive
- rather positive
- neutral
- skeptical
- negative

2.2 Do you recommend vaccinations to people of your social environment?

- yes
- no

2.3 My children are, or I would have my children vaccinated according to the Austrian National Vaccination Programme:

- yes
- no

2.4 I would …. to mandatory vaccinations for healthcare workers in hospitals, doctors’- or midwifery practices

- agree
- not agree
- – I don’t know -

2.5 I would …. to mandatory vaccinations for the attendance of state-operated facilities such as schools

- agree
- not agree
- – I don’t know -

2.6 I received knowledge on vaccinations from: (e.g. general practitioner, friends, internet…)

________________________________________________________________________________

3. Knowledge of vaccinations

3.1 It is mostly children who fall ill during current measles outbreaks.

- yes
- no
- I don’t know

3.2 WHO’s goal for the elimination of measles was the year 2015.

- yes
- no
- I don’t know

3.3 Measles can be eliminated by two MMR vaccinations and a vaccination rate of 95%.

- yes
- no
- I don’t know

3.4 The risk of encephalitis* through measles is 1 in 1,000 cases.

- yes
- no
- I don’t know

* in German, the colloquial word “Hirnentzündung” was used

3.5 Two doses of the measles vaccine (MMR) lead to lifelong protection.

- yes
- no
- I don’t know

3.6 HPV (Human Papilloma Virus): The vaccine protects from common HPV-types which may lead to cervical cancer or genital warts.

- yes
- no
- I don’t know

3.7 Women (> 18 years): Have you been vaccinated against HPV?

- Yes
  - once
  - twice
  - three times
- No

3.8 If not, why? (multiple answers possible)

- I forgot
- my doctor advised me against it
- the vaccination is not necessary
- costs
- afraid of adverse reactions
- afraid of needles
- other reasons

3.9 Is the annual cervical cancer screening at the Gynecologist’s still needed for fully HPV-vaccinated women?

4. Vaccination specific information

4.1 Do you know the Austrian National Vaccination Schedule?

- yes
- no

4.2 Do you know on what website the schedule can be found?

______________________________________________

4.3 How easy/hard is it …

… to understand why you need vaccinations?

- very hard
- hard
- easy
- very easy
- I don’t know

… to assess if information on health hazards in the media are reliable?

- very hard
- hard
- easy
- very easy
- I don’t know

… to assess which vaccinations you might need?

- very hard
- hard
- easy
- very easy
- I don’t know

… to decide if you should get vaccinated against Influenza?

- very hard
- hard
- easy
- very easy
- I don’t know

4.5 Which of the following do you consider the primary reason in the Austrian population not to get vaccinated against Influenza?

- fear of adverse reactions
- ineffective protection
- “I am not at risk”
- “the vaccination makes me ill”
- others

5. Personal vaccination status

5.1 Please cite the year in which you have last been vaccinated against the following diseases and tick off the box if you think that you are vaccinated or immune (had the disease).

- Diphtheria ___________
- Tetanus _____________
- Whooping Cough______
- Poliomyelitis__________
- Mumps ___________
- Measles __________
- Rubella ___________
- Herpes Zoster ______
- Tick-borne encephalitis______
- Influenza _________________
- HPV _____________________
- Pneumococcus ____________
- Hepatitis A ___________
- Hepatitis B _______

6. Personal attitude

6.1 How would you describe your attitude towards vaccinations?

- positive
- skeptical
- critical

6.2 What concerns do you have regarding vaccinations? We ask you to be as specific as possible. (e.g. fear of adverse reactions, fear of toxins/disease, immune system, not necessary, unnatural, …)

__________________________________________________________________________________

6.3 Would you wish for more information on the topic of vaccinations – are you sufficiently informed?

- yes
- no

If no, who should give more information? ___________________________________

6.4 What would you wish to be informed about? (e.g. information about the active agent; what vaccines are there and what for; cause and consequences of the disease; how does the vaccine work; how common are adverse reactions, …)

________________________________________________________________________________
