# Supplementary material for: Towards understanding vaccine hesitancy and vaccination refusal in Austria
Source: Wien Klin Wochenschr. 2020 Dec 11;133(13-14):703–13. doi: 10.1007/s00508-020-01777-9 (PMC8292253; doi:10.1007/s00508-020-01777-9)
Supplement: Supplementary file 2 — S2 Text. Towards understanding vaccine hesitancy. Questionnaire for the child population [file 508_2020_1777_MOESM2_ESM.docx]

Questionnaire for the children population

Introduction: Information on the project in Pöggstall and the survey

"The project „Prevent Together in Pöggstall" aims to improve vaccine protection and knowledge around their health in school children from Pöggstall. To be successful, we need your help! Please spend 2 minutes of your time and fill out the questionnaire. Your response helps „Prevent Together in Pöggstall“ to reach ist goal – for a healthier Pöggstall!“

1. Personal details

1.1 How old are you?

- <6 y.
- 6-9 y.
- 10-15y.
- >15 y.

1.2 Your sex:

- female
- male

1.3 Your education:

- primary school
- new secondary school
- grammar school (primary level)

2. General questions on vaccinations

2.1 How would you describe your general opinion on vaccinations?

- good
- rather good
- neutral
- rather skeptical
- bad

2.2 How would you describe your parents’ opinion on vaccinations?

- good
- rather good
- neutral
- rather skeptical
- bad

2.3 Do you think you have received all the scheduled vaccinations (according to the Austrian National Vaccination Schedule)?

- yes
- no

2. Do you think vaccinations should be mandatory for medical personnel in hospitals, doctors’, or midwifery practices?

- yes
- no
- I don’t know

2.5 Do you think vaccinations should be mandatory for children attending the kindergarten or school?

- yes
- no
- I don’t know

2.6 Where have you learned about vaccinations so far? (e.g. the family doctor, parents, friends, internet)

_________________________________________________________________________________

_________________________________________________________________________________

3. Questions about your current information on vaccinations

3.1 How easy/hard is it …

… to understand why you need vaccinations?

- very hard
- hard
- easy
- very easy
- I don’t know

… to assess if information on health hazards in the media are reliable?

- very hard
- hard
- easy
- very easy
- I don’t know

… to assess which vaccinations you might need?

- very hard
- hard
- easy
- very easy
- I don’t know

… to decide if you should get vaccinated against Influenza?

- very hard
- hard
- easy
- very easy
- I don’t know

4. Your knowledge of vaccination

4.1 It is important to get vaccinated to be protected from possibly severe diseases:

- yes
- no
- I don’t know

4.2 Getting vaccinated helps your body's defenses to protect you later on, by learning about sickness-causing triggers, without actually suffering from the disease”.

- yes
- no
- I don’t know

4.3 Do you know how often one should get vaccinated against Influenza?

- as soon as one got infected by it
- once a year
- as soon as somebody in their family/circle of friends got Influenza
- I don’t know

4.4 Measles are: (multiple answers possible)

- bacteria
- viruses
- cause of a highly contagious disease
- cause of a harmless children’s disease that one usually suffers from at kindergarten age
- I don’t know
- a measles infection typically includes a rash, a fever, and a cough

4.5 Against which of the following diseases do vaccines exist? (multiple answers possible)

- tonsillitis
- rabies
- mumps
- chickenpox
- hay fever
- whooping cough
- I don’t know

5. Questions on your personal attitude towards vaccinations

5.1 Would you wish for more information on the topic of vaccinations – are you sufficiently informed?

- yes
- no
  - if no, who would you like to receive more information from?
    (e.g. doctor, apothecary, nurse, midwife): _________________________

5.2 What is your personal attitude on vaccinations?

- I think positively of them
- I am rather hesitant
- I do not think well of them

5.3 What information would you wish for? (e.g. information on the active agents; what vaccines are there and what for; causes and consequences of disease; how does the vaccine work; how common are adverse reactions, …)

__________________________________________________________________________________

6. Personal vaccination status

6.1 Please give the year in which you have last been vaccinated against the following diseases:

o Diphtheria ___________

o Tetanus _____________

o Whooping Cough______

o Poliomyelitis__________

o Mumps ___________

o Measles __________

o Rubella ___________

o Tick-borne encephalitis_________

o Influenza _________________

o HPV_________________

o Pneumococcus ______________________

o Hepatitis A ___________

o Hepatitis B __________
