# Supplementary material for: Towards understanding vaccine hesitancy and vaccination refusal in Austria
Source: Wien Klin Wochenschr. 2020 Dec 11;133(13-14):703–13. doi: 10.1007/s00508-020-01777-9 (PMC8292253; doi:10.1007/s00508-020-01777-9)
Supplement: Supplementary file 3 — S3 Table vaccination rate of individual vaccines in surveyed adults, as well as OR and 95% confidence interval of having been vaccinated by age, sex, education, and knowledge score [file 508_2020_1777_MOESM3_ESM.docx]

**S3 Table vaccination rate of individual vaccines in surveyed adults, as well as OR and 95% confidence interval of having been vaccinated by age, sex, education, and knowledge score**

| Vaccination | Total % | age (years)  OR (95% CI) | sex  OR (95% CI) | education  OR (95% CI) | knowledge OR (95% CI)^a^ |
| --- | --- | --- | --- | --- | --- |
| Diphtheria | 33.5 | 16-24  1.85 (0.68-5.08) | female  0.87 (0.50-1.51) | PE  0.99 (0.42-2.35) | **1.39**  (1.18-1.63) |
|  |  | 25-39  1.91 (0.87-4.22) | male  1.0 | SLE  1.42 (0.69-2.91) |  |
|  |  | 40-60  1.73 (0.84-3.56) |  | SHE  1.11 (0.48-2.56) |  |
|  |  | 60+  1.0 |  | TE  1.0 |  |
| Tetanus | 58.7 | 16-24  1.40 (0.54-3.62) | female  **0.42** (0.24-0.73) | PE  1.55 (0.68-3.51) | **1.29**  (1.10-1.52) |
|  |  | 25-39  0.68 (0.33-1.40) | male  1.0 | SLE  1.37 (0.67-2.77) |  |
|  |  | 40-60  1.18 (0.60-2.31) |  | SHE  0.80 (0.36-1.80) |  |
|  |  | 60+  1.0 |  | TE  1.0 |  |
| Pertussis | 15.5 | 16-24  1.10 (0.29-4.19) | female  1.02 (0.48-2.15) | PE  0.59 (0.21-1.62) | **1.43**  (1.17-1.75) |
|  |  | 25-39  1.58 (0.62-4.07) | male  1.0 | SLE  0.44 (0.19-1.05) |  |
|  |  | 40-60  0.80 (0.32-1.99) |  | SHE  0.61 (0.22-1.67) |  |
|  |  | 60+  1.0 |  | TE  1.0 |  |
| Poliomyelitis | 16.6 | 16-24  2.18 (0.63-7.48) | female  0.70 (0.35-1.39) | PE  0.55 (0.18-1.67) | **1.24**  (1.02-1.51) |
|  |  | 25-39  1.26 (0.45-3.55) | male  1.0 | SLE  0.67 (0.29-1.57) |  |
|  |  | 40-60  1.88 (0.75-4.67) |  | SHE  0.79 (0.29-2.10) |  |
|  |  | 60+  1.0 |  | TE  1.0 |  |
| Measles | 30.2 | 16-24  **6.65** (2.36-18.69) | female  0.92 (0.52-1.64) | PE  1.14 (0.49-2.69) | **1.23**  (1.05-1.45) |
|  |  | 25-39  **3.72** (1.58-8.75) | male  1.0 | SLE  0.90 (0.43-1.86) |  |
|  |  | 40-60  **2.26** (1.00-5.10) |  | SHE  0.81 (0.34-1.92) |  |
|  |  | 60+  1.0 |  | TE  1.0 |  |
| Mumps | 28.1 | 16-24  **5.14** (1.85 -14.31) | female  0.88 (0.48-1.59) | PE  1.06 (0.44-2.53) | **1.44**  (1.21-1.71) |
|  |  | 25-39  **2.80** (1.21-6.47) | male  1.0 | SLE  0.86 (0.41-1.81) |  |
|  |  | 40-60  1.18 (0.53-2.62) |  | SHE  0.61 (0.25-1.50) |  |
|  |  | 60+  1.0 |  | TE  1.0 |  |
| Rubella | 31.9 | 16-24  **5.65** (1.80-17.74) | female  1.71 (0.94-3.11) | PE  0.85 (0.36-2.03) | **1.28**  (1.09-1.51) |
|  |  | 25-39  **6.21** (2.39-16.18) | male  1.0 | SLE  0.82 (0.40-1.70) |  |
|  |  | 40-60  **4.47** (1.81-11.00) |  | SHE  0.82 (0.35-1.92) |  |
|  |  | 60+  1.0 |  | TE  1.0 |  |
| TBE | 56.3 | 16-24  1.33 (0.53-3.36) | female  0.62 (0.37-1.05) | PE  0.89 (0.39-2.02) | **1.29**  (1.10-1.52) |
|  |  | 25-39  1.12 (0.54-2.32) | male  1.0 | SLE  0.66 (0.33-1.35) |  |
|  |  | 40-60  0.95 (0.49-1.85) |  | SHE  0.53 (0.24-1.20) |  |
|  |  | 60+  1.0 |  | TE  1.0 |  |
| Influenza | 10.8 | 16-24  **<0.001** (0.00-overflow) | female  **0.25**  (0.10-0.62) | PE  **0.12**  (0.02-0.68) | **1.52**  (1.18-1.97) |
|  |  | 25-39  **0.09** (0.02-0.36) | male  1.0 | SLE  0.43 (0.15-1.27) |  |
|  |  | 40-60  **0.24** (0.09-0.60) |  | SHE  0.71 (0.21-2.43) |  |
|  |  | 60+  1.0 |  | TE  1.0 |  |
| Pneumococcus | 5.1 | 16-24  2.21 (0.47-10.47) | female  0.89 (0.26-3.11) | PE  1.38 (0.30-6.31) | 1.34 (0.95-1.87) |
|  |  | 25-39  0.40 (0.09-1.71) | male  1.0 | SLE  0.23 (0.04-1.44) |  |
|  |  | 40-60  **0.08** (0.01-0.68) |  | SHE  0.62 (0.12-3.31) |  |
|  |  | 60+  1.0 |  | TE  1.0 |  |
| Hepatitis A | 20.7 | 16-24  2.68 (0.80-9.05) | female  0.55 (0.29-1.04) | PE  **0.16** (0.04-0.59) | **1.34**  (1.11-1.62) |
|  |  | 25-39  2.56 (0.95-6.93) | male  1.0 | SLE 0.58 (0.27-1.25) |  |
|  |  | 40-60  2.20 (0.88-5.49) |  | SHE  0.61 (0.25-1.52) |  |
|  |  | 60+  1.0 |  | TE  1.0 |  |
| Hepatitis B | 24.7 | 16-24  **11.93**  (3.58-39.72) | female  **0.52**  (0.28-0.98) | PE  0.46 (0.18-1.19) | **1.34**  (1.12-1.60) |
|  |  | 25-39  **4.70** (1.65-13.39) | male  1.0 | SLE  0.47 (0.22-1.00) |  |
|  |  | 40-60  **4.47** (1.66-12.01) |  | SHE  0.44 (0.18-1.10) |  |
|  |  | 60+  1.0 |  | TE  1.0 |  |
| Herpes Zoster | 1.00 | results unreliable due to n=3 | | | |
| HPV | 3.7 | 16-24  3.40 (0.25-45.95) | female  365,676,114.95 (0.00-overflow)  (only females | PE  0.35 (0.06-2.04) | 0.90 (0.60-1.33) |
|  |  | 25-39  4.37 (0.45-42.49) | male  1.0 | SLE  0.30 (0.06-1.42) |  |
|  |  | 40-60  1.63 (0.17-16.13) |  | SHE  0.49 (0.08-2.95) |  |
|  |  | 60+  1.0 |  | TE  1.0 |  |

Table 3 Vaccination rate (or immunity in the case of measles, mumps, or rubella) of surveyed adults and OR and 95% confidence interval of having been vaccinated by age, sex, education, and knowledge score (OR of having been vaccinated per single point on the knowledge score), numbers in bold are statistically significant
