# Supplementary material for: Towards understanding vaccine hesitancy and vaccination refusal in Austria
Source: Wien Klin Wochenschr. 2020 Dec 11;133(13-14):703–13. doi: 10.1007/s00508-020-01777-9 (PMC8292253; doi:10.1007/s00508-020-01777-9)
Supplement: Supplementary file 4 — S4 Table vaccination rate of individual vaccines in surveyed children, as well as OR and 95% confidence interval of having been vaccinated by age, sex, parental skepticism, and knowledge score [file 508_2020_1777_MOESM4_ESM.docx]

**S4 Table vaccination rate of individual vaccines in surveyed children, as well as OR and 95% confidence interval of having been vaccinated by age, sex, parental skepticism, and knowledge score**

| Vaccination | Total % | age (years)  OR (95% CI) | sex  OR (95% CI) | parental attitude  OR (95% CI) | knowledge OR (95% CI)^a^ |
| --- | --- | --- | --- | --- | --- |
| Diphtheria | 39.9 | 6-9  1.57 (0.89-2.78) | female  **2.31** (1.43-3.73) | positive  1.14 (0.60-2.14) | **1.19**  (1.07-1.32) |
|  |  | 10+  1.0 | male  1.0 | negative  1.0 |  |
| Tetanus | 45.9 | 6-9  1.35 (0.77-2.36) | female  **1.69** (1.06-2.70) | positive  1.24 (0.67-2.30) | **1.16**  (1.05-1.29) |
|  |  | 10+  1.0 | male  1.0 | negative  1.0 |  |
| Pertussis | 23.4 | 6-9  **2.42** (1.32-4.47) | female  **1.93** (1.11-3.36) | positive  1.28 (0.60-2.73) | **1.19**  (1.05-1.34) |
|  |  | 10+  1.0 | male  1.0 | negative  1.0 |  |
| Poliomyelitis | 25.0 | 6-9  **0.46** (0.22-0.96) | female  1.31 (0.76-2.24) | positive  1.22 (0.57-2.61) | **1.24**  (1.10-1.40) |
|  |  | 10+  1.0 | male  1.0 | negative  1.0 |  |
| Measles | 42.7 | 6-9  1.27 (0.72-2.25) | female  **1.65** (1.02-2.65) | positive  1.16 (0.61-2.19) | **1.25**  (1.12-1.39) |
|  |  | 10+  1.0 | male 1.0 | negative  1.0 |  |
| Mumps | 41.5 | 6-9  1.27 (0.72-2.25) | female  1.61 (1.00-2.59) | positive  1.64 (0.85-3.17) | **1.24**  (1.11-1.38) |
|  |  | 10+  1.0 | male  1.0 | negative  1.0 |  |
| Rubella | 38.6 | 6-9  1.15 (0.65-2.05) | female  1.44 (0.89-2.33) | positive  1.37 (0.71-2.65) | **1.24**  (1.11-1.38) |
|  |  | 10+  1.0 | male  1.0 | negative  1.0 |  |
| TBE | 44.9 | 6-9  1.47 (0.83-2.61) | female  1.38 (0.86-2.21) | positive  **3.25** (1.63-6.47) | **1.14** (1.03-1.26) |
|  |  | 10+  1.0 | male  1.0 | negative  1.0 |  |
| Influenza | 2.2 | 6-9  0.74 (0.08-6.42) | female  2.24 (0.40-12.62) | positive  1.26 (0.14-11.64) | 1.02 (0.72-1.45) |
|  |  | 10+  1.0 | male  1.0 | negative  1.0 |  |
| HPV | 7.0 | 6-9  0.15 (0.02-1.18) | female  **4.62** (1.62-13.16) | positive  1.65 (0.45-6.07) | 1.18 (0.98-1.44) |
|  |  | 10+  1.0 | male 1.0 | negative  1.0 |  |
| Pneumococcus | 11.1 | 6-9  **2.68** (1.24-5.82) | female  1.13 (0.55-2.36) | negative  2.13 (0.61-7.48) | **1.28** (1.09-1.52) |
|  |  | 10+  1.0 | male  1.0 | positive  1.0 |  |
| Hepatitis A | 7.6 | 6-9  0.52 (0.15-1.79) | female  0.79 (0.34-1.86) | positive  1.40 (0.39-5.05) | 1.06 (0.89-1.28) |
|  |  | 10+  1.0 | male  1.0 | negative  1.0 |  |
| Hepatitis B | 25.6 | 6-9  1.22 (0.65-2.27) | female  **1.88** (1.11-3.18) | positive  1.53 (0.73-3.22) | **1.15** (1.03-1.29) |
|  |  | 10+  1.0 | male  1.0 | negative  1.0 |  |

Vaccination rate of surveyed children of individual vaccines, as well as OR and 95% confidence interval of having been vaccinated by age, sex, parental skepticism, and single point on the knowledge score, numbers in bold were statistically significant
