# Supplementary material for: Towards understanding vaccine hesitancy and vaccination refusal in Austria
Source: Wien Klin Wochenschr. 2020 Dec 11;133(13-14):703–13. doi: 10.1007/s00508-020-01777-9 (PMC8292253; doi:10.1007/s00508-020-01777-9)
Supplement: Supplementary file 5 — S5 Table OR and 95% confidence interval of skeptical and negative attitude/vaccine recommendation/getting one’s children vaccinated/agreement to mandatory vaccination for state-operated institutions/agreement to mandatory vaccination for HCW by age, sex, education, and knowledge score [file 508_2020_1777_MOESM5_ESM.docx]

|  | Total % | age  OR (95% CI) | sex  OR (95% CI) | education  OR (95% CI) | knowledge OR (95% CI)^a^ |
| --- | --- | --- | --- | --- | --- |
| **skeptical and negative attitude** | 21.0 | 16-24  1.73 (0.57-5.28) | female  1.11 (0.59-2.10) | PE  0.89 (0.30-2.60) | **0.63** (0.50-0.79) |
|  |  | 25-39  1.38 (0.53-3.61) | male  1.00 | SLE  1.04 (0.41-2.63) |  |
|  |  | 40-60  1.83 (0.75-4.45) |  | SHE  1.84 (0.67-5.02) |  |
|  |  | 60+  1.00 |  | TE  1.00 |  |
| **recommendation** | 55.6 | 16-24  0.93 (0.36-2.40) | female  0.70 (0.40-1.22) | PE  0.83 (0.34-1.99) | **1.66**  (1.39-2.00) |
|  |  | 25-39  0.71 (0.33-1.51) | male  1.00 | SLE  0.55 (0.25-1.18) |  |
|  |  | 40-60  1.18 (0.59-2.37) |  | SHE  **0.34** (0.14-0.82)) |  |
|  |  | 60+  1.00 |  | TE  1.00 |  |
| **children vaccinated** | 73.2 | 16-24  0.56 (0.20-1.54) | female  0.80 (0.44-1.43) | PE  1.27 (0.49-3.27) | **1.34** (1.11-1.63) |
|  |  | 25-39  0.51 (0.22-1.17) | male  1.00 | SLE  0.83 (0.37-1.85) |  |
|  |  | 40-60  0.77 (0.35-1.70) |  | SHE  0.77 (0.31-1.92) |  |
|  |  | 60+  1.00 |  | TE  1.00 |  |
| **general mandatory**  **vaccination for attendance of**  **state-operated institutions** | 39.3 | 16-24  **0.16** (0.05-0.55) | female  0.78 (0.45-1.36) | PE  1.00 (0.43-2.30) | **1.46**  (1.24-1.73) |
|  |  | 25-39  **0.46** (0.22-0.97) | male  1.00 | SLE  0.81 (0.39-1.66) |  |
|  |  | 40-60  **0.51** (0.26-0.99) |  | SHE  0.48 (0.20-1.15) |  |
|  |  | 60+  1.00 |  | TE  1.00 |  |
| **mandatory vaccination**  **HCW** | 54.2 | 16-24  0.88 (0.35-2.22) | female  1.01 (0.60-1.72) | PE  0.66 (0.28-1.54) | **1.36**  (1.16-1.60) |
|  |  | 25-39  0.50 (0.24-1.04) | male  1.00 | SLE  **0.41**  (0.19-0.85) |  |
|  |  | 40-60  0.80 (0.41-1.56) |  | SHE  **0.39**  (0.17-0.91) |  |
|  |  | 60+  1.00 |  | TE  1.00 |  |

**S5 Table OR and 95% confidence interval of skeptical and negative attitude/vaccine recommendation/getting one’s children vaccinated/agreement to mandatory vaccination for state-operated institutions/agreement to mandatory vaccination for HCW by age, sex, education, and knowledge score**

S3 Table: OR and 95% confidence interval of skeptical and negative attitude/vaccine recommendation/getting one’s children vaccinated/agreeing to mandatory vaccination for state-operated institutions/agreeing to mandatory vaccination for HCW by age, sex, education, and knowledge score. NS= not significant (p>0.05). PE= primary education, SLE= secondary lower education, SHE=secondary higher education, TE= tertiary education
