# Supplementary material for: Towards understanding vaccine hesitancy and vaccination refusal in Austria
Source: Wien Klin Wochenschr. 2020 Dec 11;133(13-14):703–13. doi: 10.1007/s00508-020-01777-9 (PMC8292253; doi:10.1007/s00508-020-01777-9)
Supplement: Supplementary file 6 — S6 Table OR and 95% confidence interval of a general skeptical or negative attitude/all vaccinations received/agreement on mandatory vaccination school attendance/agreement to mandatory vaccinations for HCW in children by age, sex, parents’ opinion, and knowledge score [file 508_2020_1777_MOESM6_ESM.docx]

**S6 Table OR and 95% confidence interval of a general “skeptical” or “negative” attitude/all vaccinations received/agreement on mandatory vaccination school attendance/ agreement to mandatory vaccinations for HCW in children by age, sex, parents’ opinion, and knowledge score**

|  | Total % | age  OR (95% CI) | sex  OR (95% CI) | parent’s opinion | knowledge OR (95% CI)^a^ |
| --- | --- | --- | --- | --- | --- |
| **general opinion: skeptical/negative** | 17.4 | 6-9  **2.51** (1.04-6.05) | female  0.77 (0.35-1.72) | positive/rather p. **0.04** (0.02-0.09) | **0.77** (0.66-0.91) |
|  |  | 10+  **1.00** | male  1.00 | skeptical/negative **1.00** |  |
| **parents skeptical/negative opinion** | 18.4 | 6-9  1.26 (0.64-2.50) | female  **2.09** (1.16-3.78) |  | - |
|  |  | 10+  1.00 | male  **1.00** |  |  |
| **all vaccinations received** | 63.0 | 6-9  1.36 (0.73-2.56) | female  0.97 (0.58-1.61) | positive **3.86** (2.02-7.37) | **1.28** (1.14-1.43) |
|  |  | 10+  1.00 | male  1.00 | skeptical/negative **1.00** |  |
| **mandatory vaccination**  **school attendance** | 30.7 | 6-9  1.13 (0.61-2.12) | female  0.64 (0.38-1.08) | positive **13.33** (3.15-56.42) | **1.16**  (1.03-1.30) |
|  |  | 10+  1.00 | male | skeptical/negative **1.00** |  |
| **mandatory vaccination**  **HCW** | 40.2 | 6-9  0.99 (0.54-1.81) | female  0.93 (0.56-1.52) | positive **6.39** (2.58-15.84) | **1.28**  (1.14-1.44) |
|  |  | 10+  1.00 | male  1.00 | skeptical/negative **1.00** |  |

S4 Table OR and 95% confidence interval of general and personal “skeptical” or “negative” attitude/all vaccinations received/agreeing on mandatory vaccination school attendance/ agreeing to mandatory vaccinations for HCW by age, sex, parents’ opinion, and single, NS= not significant (p>0.05)
